# A clinical trial about effects of prebiotic and probiotic supplementation on weight loss, psychological profile and metabolic parameters in obese subjects

**DOI:** 10.1002/edm2.402

**Published:** 2023-01-06

**Authors:** Rym Ben Othman, Nadia Ben Amor, Faten Mahjoub, Olfa Berriche, Chaima El Ghali, Amel Gamoudi, Henda Jamoussi

**Affiliations:** ^1^ Faculty of Medicine of Tunis Institute National de Nutrition et de Technologie Alimentaire de Tunis and University of Tunis el Manar Tunis Tunisia

**Keywords:** obesity, prebiotics, probiotics, weight loss

## Abstract

**Introduction:**

The management of obesity is difficult with many failures of lifestyle measures, hence the need to broaden the range of treatments prescribed. The aim of our work was to study the influence of pre and probiotics on weight loss psychological profile and metabolic parameters in obese patients.

**Methods:**

It is a clinical trial involving 45 obese patients, recruited from the Obesity Unit of the National Institute of Nutrition between March and August 2022 divided into three groups: diet only (low‐carbohydrate and reduced energy diet), prebiotics (30 g of carob/day) and probiotics (one tablet containing *Bifidobacterium longum*, *Lactobacillus helveticus*, *Lactococcus lactis*, *Streptococcus thermophilus/*day). The three groups were matched for age, sex and BMI. Patients were seen after 1 month from the intervention. Anthropometric measures, biological parameters, dietary survey and psychological scores were performed.

**Results:**

The average age of our population was 48.73 ± 7.7 years, with a female predominance. All three groups showed a significant decrease in weight, BMI and waist circumference with *p* < .05. Only the prebiotic and probiotic group showed a significant decrease in fat mass (*p* = .001) and a significant increase in muscle strength with *p* = .008 and .004, but the differences were not significant between the three groups. Our results showed also a significant decrease in insulinemia and HOMA‐IR in the prebiotic group compared to the diet‐alone group (*p* = .03; *p* = .012) and the probiotic group showed a significant decrease in fasting blood glucose compared to the diet alone group (*p* = .02). A significant improvement in sleep quality was noted in the prebiotic group (*p* = .02), with a significant decrease in depression, anxiety and stress in all three groups.

**Conclusions:**

The prescription of prebiotics and probiotics with the lifestyle measures seems interesting for the management of obesity especially if it is sarcopenic, in addition to the improvement of metabolic parameters and obesity‐related psychiatric disorders.

## Graphical abstract

The supplementation with prebiotics and probiotics showed an improvement in lean mass, glycaemic profile, insulin resistance and uric acid more than diet alone.

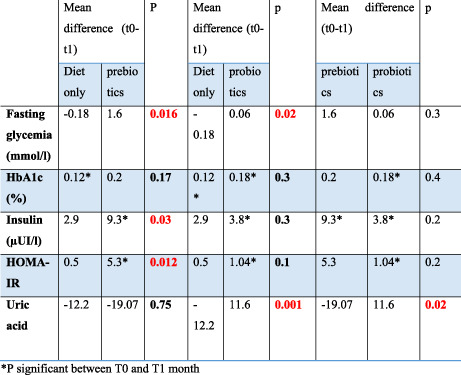

## 1 INTRODUCTION

Today, obesity is a global epidemy according to the World Health Organization, given the increase in its frequency in the world, and its responsibility in the appearance of several chronic pathologies, such as type 2 diabetes, hypertension, cardiovascular diseases, respiratory diseases, osteoarticular diseases, cancer and other pathologies.

In 2021, the WHO announced that more than 40% of men and women, or 2.2 billion people, are overweight and that an unbalanced diet was responsible for at least 8 million deaths per year. It is estimated that by 2025, 167 million people would be at risk of impaired health due to obesity.^1^

In Tunisia, the prevalence of obesity was 26.2% in 2016 according to the results of the “Tunisian Health Examination Survey‐2016”.^2^

This disease is multifactorial, among the contributing factors of obesity are: a high‐fat diet, a sedentary lifestyle, but also the imbalance of the intestinal flora, “the gut microbiota”^3^ which today represents the focus of several publications.

Gut microbiota is defined by all the beneficial microorganisms that live and grow in the intestine. It is set up from birth and evolves according to different factors such as antibiotic treatments or diet (presence of fibres, richness of foods in pre and probiotics). Probiotics are living microorganisms, they are bacteria such as *Lactobacilli*, *Bifidobacteria*, *Streptococci* and many others or yeasts. They can be present naturally in our diet, especially in fermented foods such as certain yoghurts or fermented milks, whereas prebiotics represent substrates for these bacteria which allow them to ensure their growth and thus exercise their beneficial roles, they are also provided by our diet, from the dietary fibres present in vegetables and fruits, such as carob, chicory and others.

Today, the microbiota is considered a therapeutic revolution, where researchers use its enrichment to prevent or treat certain diseases including obesity,^4^ such as faecal transplantation,^5^ but also the enrichment of the microbiota by prebiotics and probiotics to treat obesity.^6^, ^7^ Hence, our interest in transposing these theoretical results to clinical practice.

Aim: The objective of this interventional clinical trial was to evaluate the effects of a probiotic supplement containing *Bifidobacteruim*, *Lactobacillus* strains and a prebiotic supplement by carob on the changes in body composition and metabolic biomarkers in subjects with obesity (main purpose), we also checked the psychological profile of the population (quality of sleep, stress, anxiety and depression) as secondary purpose.

## 2 MATERIALS AND METHODS

We conducted a prospective interventional study at the obesity unit at the Zouhair El Kallel National Institute of Nutrition and Food Technology of Tunis, from March 2022 to August 2022.

We included in our study obese patients (BMI ≥30 kg/m^2^) aged over 18 years. Patients with: renal failure, hypothyroidism, cancer, diabetic patients on insulin, on long‐term corticosteroid therapy, former patients of the obesity unit were not included. No participants dropped out of the study during the intervention period.

Forty‐five patients were recruited on their first visit to the obesity unit (T0) and were randomly assigned to three groups matched for age, sex and BMI. All participants were enrolled in the weight loss program at the beginning of the study and followed a low‐carbohydrate, reduced‐energy intake eating plan provided by the same dietician.

- First group called “diet only”: on low‐calorie diet alone without any intervention (15 patients).
- Second group: 15 patients on the same diet plan but additionally received prebiotic supplementation (2 carob beans/day about 30 g) called “prebiotic group”.
- Third group: same diet with probiotic supplementation (*n* = 15). The probiotic component used in the study was one tablet containing an association of four microbiological strains which are: *Bifidobacteruim longum*, *Lactobacillus helveticus*, *Lactococcus lactis*, *Streptococcus thermophilus* (1 tablet (10.10^9^ UFC/capsule)/day) called “probiotic group”. The probiotic supplement was produced by Pileje Labs.

Patients were reassessed after 1 month (T1) and we track adherence by regular phone calls.

All subjects gave their informed consent for participating in the study. The study was approved by the ethical committee of the national institute of nutrition of Tunis and the clinical trial was registered under number PACTR202210705998795 in the Pan African Clinical Trial Registry.

Body mass index (BMI) was calculated using body weight and height measured with bare feet and in minimal clothing according to the World Health Organization definition and classification.^8^ Body composition parameters (body fat mass and percentage and body lean mass) were acquired before and after 1 month of intervention by impedance meter TANITA BC418MA.

We took the waist circumference of the patients. Muscle strength was measured by the handgrip. Sarcopenia was defined by muscle strength lower than 27 kg for men and 16 for women.

A biological assessment was carried out at T0 and T1 including: fasting glycaemia, HbA1C, Cholesterol, triglycerides, HDL, calculated LDL Friedwald formula,^9^ insulinemia, calculated HOMA‐IR (HOMA‐IR = (insulin (mU/l) x glycaemia (mmol/l))/22.5), AST, ALT, GGT, creatinine and calculated eGFR.

Blood glucose results were interpreted according to American diabetes association guidelines.^10^

We looked at the physical examination for blood pressure and other complications of obesity such as hernia, sleep apnoea syndrome, osteoarthritis and NASH and if necessary we completed with the necessary radiological examinations.

All patients benefited from an interview including food survey, stress questionnaire (Cunji), sleep questionnaire (Epworth), symptoms of depression and anxiety (HADS).

For the evaluation of stress, we used the brief stress evaluation scale, this is the scale of Cungi 1997.^11^ This scale is made up of 11 items, and for each the response is from 1 to 6.

The evaluation of the quality of sleep was carried out using the Epworth Sleepiness Scale,^12^ this questionnaire assesses the level of daytime sleepiness of the patient. It is composed of eight items, and for each situation, the patient must select an answer from (0 to 3). The interpretation is as follows:

- A total of less than 10 suggests that there is no excessive daytime sleepiness.
- A total of 10 and above suggests excessive daytime sleepiness.

To assess the depressive state of the patients, we used the “HAD” scale (Hospital Anxiety and Depression Scale).^13^ This is a structured questionnaire of 14 items. This questionnaire consists of two subscales, each having 7 items, one for anxiety, the other for depression. Each item is rated on a 4‐point scale, that is from 0 to 3, evaluating the intensity of symptoms over the past week. The scores therefore range from 0 to 21 and the highest scores correspond to the presence of more severe symptoms. The addition of the scores obtained for each item allows the following interpretation:

- Less than 7 points: no symptoms of depression.
- Eight to 10 points: doubtful symptomatology.
- Eleven and over: certain symptomatology.

### 2.1 Statistical analysis

The three‐variable ANOVA with Student's *t* test for paired series were used for group comparison of the body composition and metabolic parameters at T1 and T0 (SPSS Statistics, v. 25). The results were expressed as mean ± SD, and mean differences were considered significant at *p* < .05.

## 3 RESULTS

The average age of our population was 48.73 ± 7.7 years with extremes ranging from 33 to 63 years. Half of the population (51%) was over 50 years old. The majority of participants were female 93.3% (*n* = 42) against 6.7% (*n* = 3) of men. Past medical history, complications and lab test results are present in Table 1.

**TABLE 1:** Past medical history, complications of obesity and lab test results

	Diet only (%)	Prebiotic (%)	Probiotic (%)	*p*
Past medical history				
Diabetes	6.7	26.7	13.3	.3
Hypertension	6.7	20	33.3	.2
Dyslipidaemia	6.7	26.7	13.3	.3
Active smokers (%)	6.7	13.3	6.7	.7
Osteoarthritis (%)	33.3	20	26.7	.6
Sleep apnoea syndrome (%)	26.7	66.7	33.3	.07
Hernia (%)	13.3	6.7	13.3	.6
NASH (%)	24	30	24	.42
Diabetes (%)	13.3	53.3	20	.06
Prediabetes (%)	13.3	6.7	33.3	.06
Insulin resistance (%)	6.7	0	7.1	.65
High TG levels (%)	46.7	66.7	26.7	.18
Low HDL levels(%)	46.7	46.7	33.3	.27
High LDL levels(%)	26.7	33.3	33.3	.4

Blood pressure values are comparable in the three groups.

Our three groups were matched for BMI. There was no statistically significant difference for anthropometric measurements (weight, height, IMC, fat mass, muscle mass and waist circumference) between the three groups. In addition, the majority of patients in all three groups had normal muscle strength. Sarcopenia at T0 was noted in 20% in the diet‐only group, 6.7% in the prebiotic group and 13.3% in the probiotic group.

In each group, 93.3% of patients were sedentary.

At recruitment, we performed a frequency questionnaire consumption of foods rich in prebiotics and probiotics such as coffee, tea, garlic, onion, fermented foods, cacao, yoghurts and fruits. There were no differences between groups.

No patient reported alcohol consumption and none had a regular consumption of carob.

Most of the patients of the three groups had a high level of anxiety, depression and stress but without statistically significant difference.

The result of the intervention after 1 month are in Table 2.

**TABLE 2:** Results of the intervention on different parameters for the three groups

	Diet only			Prebiotic			Probiotic		
	T0	T1	*p*	T0	T1	*p*	T0	T1	*p*
Weight (kg)	103.7	101.2	**.001**	103.5	101.6	**.003**	106.09	104.4	**.02**
Fat mass (kg)	46.7	44.8	**.07**	47.3	44.3	**.001**	47.5	45.01	**.001**
Lean mass (kg)	54.06	53.5	**.3**	55.1	55.6	.2	55.5	56.4	**.08**
Waist circumference (cm)	119	117.3	**.01**	124	120	**.03**	122	119	**.001**
Muscle strength (kg)	24.4	24.3	**.8**	27.4	28.8	**.008**	24.8	26.5	**.004**
Systolic blood pressure (mmHg)	13	12.8	**.6**	13	12.2	**.03**	13.3	12.6	**.01**
Fasting glucose (mmol/l)	5.3	5.5	**.27**	7.5	5.1	**.2**	5.66	5.6	**.6**
HbA1c (%)	5.6	5.5	**.03**	6.6	6.3	**.3**	5.8	5.6	**.003**
Insulin (μUI/l)	18.4	15.2	**.07**	23.8	14.5	**.002**	17.5	13.7	**.005**
HOMA‐IR	4.3	3.8	**.2**	9.1	3.8	**.009**	4.5	3.4	**.009**
Cholesterol (mmol/l)	5.2	4.8	**.03**	5.3	4.9	**.005**	5.2	4.6	**.08**
HDL (mmol/l)	1.6	1.05	**.9**	1.07	1.08	**.8**	1.2	1.22	**.7**
LDL (mmol/l)	3.2	2.9	**.05**	3.3	2.9	**.003**	3.2	2.8	**.004**
Triglycerides (mmol)	1.7	1.6	**.4**	1.9	1.4	**.001**	1.6	1.4	**.03**
ALAT (UI/l)	21.2	18.4	**.03**	21.3	20.8	**.7**	21.1	17.8	**.01**
Uric acid	277.2	289.4	**.4**	346.3	365.3	**.3**	295.7	284.2	**.1**
Epworth	9.8	8.6	**.06**	8.7	7	**.02**	10.2	7.9	**.03**
Anxiety	13.3	11.2	**.02**	11.4	9.4	**.01**	13.3	11.6	**.06**
depression	12.4	9.9	**.001**	11.2	8.06	**.01**	11.5	8.9	**.001**
Stress	40.1	33.4	**.01**	36.2	31.3	**.001**	35.6	29.3	**.002**

Bold value indicates statistically significant *p* < 0.05.

The results of anthropometric measurements after the intervention in the three groups showed a statistically significant decrease in weight, BMI and WC, but muscle strength has increase only with pre and probiotics.

The population has significantly decreased energy and macronutrient (protein, carbohydrate and lipid) intake, with a significant decrease in sugar and sodium intake.

A significant increase in fibre intake was noted in the diet and prebiotic group but not in the probiotic group. The quality of sleep was not improved by the diet only and probiotics did not enhance anxiety.

Taking probiotics was associated with the occurrence of diarrhoea in 20% of cases (*p* < .001).

Then we compared the diet alone versus prebiotics group for all the parameters listed in Table 3. The difference was not significant. Then it was the diet alone group versus probiotics and finally prebiotics versus probiotics.

**TABLE 3:** Comparison of biological assessments according to the intervention

	Mean difference (T0–T1)		*p*	Mean difference (T0–T1)		*p*	Mean difference (T0–T1)		*p*
	Diet only	Prebiotics		Diet only	Probiotics		Prebiotics	Probiotics	
Fasting glycaemia (mmol/l)	−0.18	1.6	**.016**	−0.18	0.06	**.02**	1.6	0.06	.3
HbA1c (%)	0.12^a^	0.2	**.17**	0.12^a^	0.18^a^	**.3**	0.2	0.18^a^	.4
Insulin (μUI/l)	2.9	9.3^a^	**.03**	2.9	3.8^a^	**.3**	9.3^a^	3.8^a^	.2
HOMA‐IR	0.5	5.3^a^	**.012**	0.5	1.04^a^	**.1**	5.3	1.04^a^	.2
Uric acid	−12.2	−19.07	**.75**	−12.2	11.6	**.001**	−19.07	11.6	**.02**

Bold value indicates statistically significant *p* < 0.05.

^a^ *p*significant between T0 and T1 month.

Our conclusion is that the different therapeutic means are equal for the dietary survey, the different scores (stress, sleep, anxiety and depression).

The influence of the three means on weight loss is equivalent even if it is the diet alone group which reduced the weight more except for the lean mass which was clearly increased by probiotics compared to diet (*p* = .05). On the other hand, significant differences between the three means were found in the results of the blood tests represented in Table 3.

Prebiotics and probiotics were better than diet for the reduction of fasting glycemia and insulin resistance but probiotics did not lower uric acid as much as others.

## 4 DISCUSSION

This study was an interventional clinical trial designed to examine the effects of a combination of probiotic bacteria *B. longum*, *L. helveticus*, *L. lactis*, *S. thermophilus* and a prebiotic supplement by 30 g/day of carob on changes in body composition, metabolic biomarkers and psychological profile in obese human subjects enrolled on a weight loss program. The weight loss program was a low‐carbohydrate, energy‐restricted eating plan. The study has confirmed that a low‐carbohydrate, restricted‐energy diet can be effectively used for weight loss in obese individuals.

Our work has some strength—to our knowledge in Tunisia no one studied the association between prebiotics or probiotics and obesity, the only Tunisian study that has worked on the microbiota has studied the imbalance of the microbiota in diabetic patients.^14^ The use of carob as a prebiotic for weight loss is an innovation that fits into abandoned Tunisian habits. Carob is available at a nominal cost less than some fruits and vegetables. Our study focused on several parameters apart from anthropometry, such as biology and other assessment tests such as the Epworth score, the HAD and the Cungi stress score but it has some limitations like the small number of patients for each group and microbiological analysis for the gut microbia was not performed. In addition, the study was conducted over a month; perhaps a longer duration of intervention would show other results.

Many studies have shown the effect of pre or probiotic on the weight loss. Sergeev et al.,^15^ compared the effect of symbiotic supplementation (prebiotic and probiotic) on the body composition of obese patients against a placebo group which received only a low‐calorie diet, they found a significant decrease in weight in both groups. However, the study of Hiel et al.,^16^ using inulin as prebiotic compared to placebo, found a significant reduction in weight in the prebiotic group. This difference may be due to the difference in the prescribed diet and also to the difference in the number of patients. In addition, the study by Stenman et al.,^17^ which is a study that compared the effect of prebiotic alone, probiotic alone and prebiotic+probiotic to a placebo group, found that only the probiotic alone group presented weight loss compared to the other groups. Some other studies did not found a difference between groups.^18^, ^19^ This difference may be due to the difference in the diet given and also the type of prebiotic and probiotic used. Similarly, Rodriguez in their studies showed that there were responders and non‐responders in obese patients treated with prebiotics depending on the initial species of intestinal flora present in the host during the intervention.^20^

Indeed, the microbiota intervenes in the regulation of energy expenditure by acting on specific hormones, thanks to a bidirectional signalling between the brain and the intestine, the gut microbiota regulates appetite and energy expenditure then follows a weight regulation.^21^ Prebiotics act on the microbiota by increasing the production of short‐chain fatty acids, which in turn causes a cascade of modifications leading to weight reduction and improved metabolic parameters.^22^

Our study showed a significant increase in muscle strength in both the prebiotic group and the probiotic group. As well as Zahao and Kang in their studies.^23^, ^24^ Alteration of the gut microbiota has been shown to directly affect muscle strength. Probiotics, prebiotics and short‐chain fatty acids are potential new therapies to improve lean mass and physical performance. Strains of *Lactobacillus* and *Bifidobacterium* (present in Lactibiane*) can restore age‐related muscle loss. The pathways by which microbiota influence muscle are diverse and complex.^25^

Our results showed a beneficial effect of prebiotics and probiotics on carbohydrate metabolism. These results were in agreement with the study conducted by Miller et al.,^26^ which found that the symbiotic yoghurt protected mice against diabetes by significantly improving fasting blood glucose levels versus unenriched control yoghurt. In addition, a preparation rich in fibre and lactulose as prebiotics used in an old clinical study,^27^ showed a decrease in blood sugar in 10 obese patients.

Oral supplementation with prebiotics and probiotics acts on the regulation of glycaemia, the mechanism of action consists in reducing the secretion of inflammatory markers such as IFN‐γ and IL‐1β by increasing the production of IL‐10 anti‐inflammatory. In addition, probiotics stimulate the secretion of the neurotransmitter GABA which decreases the production of glucagon and stimulates the production of insulin.^28^, ^29^

Our study showed a decrease in uric acid in the probiotic group with a significant difference compared to the diet‐alone group and the prebiotic group. To study the effect of probiotics on uric acid, there was first the pilot study of Garcia‐Arroyo carried out in 2018 on six rats which affirmed this hypothesis.^30^ Then other studies followed with the same results.^31^, ^32^

The decrease in energy intake found after prebiotic and probiotic supplementation is explained by the stimulation of leptin secretion and the decrease in ghrelin secretion, which increase satiety and consequently decrease in intake. In addition, the reduction of microbiome lipopolysaccharides by pre and probiotics promotes reduced appetite by increasing satiety.^33^

A decrease in Epworth score was found in all three groups. Our study was consistent with others.^34^, ^35^ However, the study by Buigues et al.^36^ did not show conclusive results of prebiotics on sleep quality. Following the fermentation of fibres from prebiotics by microbiota, there will be production of butyrate which improves sleep quality^37^ but the mechanisms involved are more complex than that.^38^

The three means were comparable in their influence on depression and anxiety. Other studies proved a good improvement of these symptoms when patients took probiotic.^39^, ^40^ It has been shown that probiotics stimulate the production of inhibitory neurotransmitters such as the neurotransmitter GABA, which causes a reduction in anxiety and depression.^41^ On the other hand, the imbalance of the gut microbiota is responsible for the occurrence of depression by the decrease in the production of some lipid metabolites (endogenous cannabinoids).^42^

As for the stress, prebiotics and probiotics increase the production of serotonin, which is a molecule involved in mood regulation, by stimulating the synthesis of tryptophan^43^ which improves the symptoms of stress.

## 5 CONCLUSION

The imbalance in the functioning of the body is due on the one hand to the imbalance of the gut microbiota because of obesity which alters the beneficial microorganisms and on the other hand this alteration which further promotes obesity by several mechanisms and signalling pathways.^44^

The intestinal microbiota, as it is called the second brain, intervenes in the regulation of the functioning of the organism, which has been demonstrated by several studies. Hence the importance of modulating the gut microbiota with prebiotics and probiotics to treat obesity and improve related metabolic parameters.

In the light of this study and other studies, it is advisable to take certain measures to treat obesity:

- Follow a diet balanced in energy intake to prevent the alteration of the gut microbiota.
- Enrich the diet with foods rich in prebiotics and probiotics, either to prevent the onset of obesity or to treat it.
- Treatment with pre and probiotics should be considered in case of sarcopenic obesity.
- Adopt treatment with prebiotics and probiotics, especially if obesity is linked to a glycaemic disorder.
- Prescription of prebiotics and probiotics can Improve the quality of sleep, anxiety and stress in some cases.

## AUTHOR CONTRIBUTIONS

**Nadia Ben Amor:** Visualization (equal). **Faten Mahjoub:** Visualization (equal). **Olfa Berriche:** Visualization (equal). **Chaima El Ghali:** Investigation (equal). **Amel Gamoudi:** Project administration (equal). **Henda Jamoussi:** Writing – review and editing (equal).

## FUNDING INFORMATION

This research received no funding.

## CONFLICT OF INTEREST

The authors declare no conflict of interest.

## Data Availability

Data sharing is not applicable to this article as no new data were created or analyzed in this study.
